# The Role of Inflammatory Markers in Linking Metabolic Syndrome to Cognitive Decline in Middle-Aged Women: A Focus on TNF-α and IL-6

**DOI:** 10.3390/metabo15030186

**Published:** 2025-03-11

**Authors:** Kinga Mruczyk, Angelika Cisek-Woźniak, Marta Molska, Aleksandra Skoczek-Rubińska

**Affiliations:** Department of Dietetics, Faculty of Physical Culture in Gorzów Wielkopolski, Poznan University of Physical Education, Estkowskiego 13, 66-400 Gorzów Wielkopolski, Poland; a.cisek@awf-gorzow.edu.pl (A.C.-W.); m.molska@awf-gorzow.edu.pl (M.M.); a.skoczek-rubinska@awf-gorzow.edu.pl (A.S.-R.)

**Keywords:** metabolic syndrome, insulin resistance, Mini-Mental State Examination, MMSE, inflammation, cognitive function

## Abstract

**Background:** Metabolic syndrome (MetS) and related disorders, such as insulin resistance, pose significant health risks in middle-aged women, including cognitive decline. Chronic inflammation, characterized by elevated levels of interleukin-6 (IL-6) and tumour necrosis factor-alpha (TNF-α), has been identified as a key mechanism linking metabolic disturbances to neurodegenerative processes. **Methods**: This study aimed to examine the associations between metabolic disorders, inflammatory markers, and cognitive function among middle-aged women. A cross-sectional study was conducted on 179 non-smoking perimenopausal and postmenopausal women aged 43–73 years. Anthropometric, metabolic, and cognitive parameters were assessed, including body mass index (BMI), waist-to-height ratio (WHtR), fasting glucose (GLU), triglycerides (TG), IL-6, TNF-α, and Mini-Mental State Examination (MMSE) scores. Logistic regression models were applied to evaluate the relationships between inflammation, MetS components, and cognitive impairments. **Results**: Women with insulin resistance showed significantly worse metabolic profiles and lower MMSE scores (23.98 vs. 24.91, *p* = 0.032). IL-6 levels were strongly associated with hypertriglyceridemia (OR = 1.096, 95% CI: 1.044–1.151, *p* < 0.001) and insulin resistance (OR = 1.068, 95% CI: 1.030–1.107, *p* < 0.001), while TNF-α correlated with abdominal obesity (WHtR OR = 1.429, 95% CI: 1.005–2.031, *p* = 0.047). Moreover, TNF-α was a significant predictor of cognitive impairments (OR = 1.362, 95% CI: 1.153–1.610, *p* < 0.001), whereas IL-6 showed no significant association. **Conclusions**: These findings highlight that TNF-α may be a key inflammatory marker associated with metabolic disturbances and cognitive decline in middle-aged women. IL-6 appears to be more specifically linked to lipid abnormalities and insulin resistance. Targeted interventions to reduce inflammation may moderate metabolic and cognitive risks in this population.

## 1. Introduction

The increasing prevalence of metabolic disorders among middle-aged women represents a significant public health concern due to their association with a range of chronic conditions, including cardiovascular diseases, diabetes, and a decline in cognitive function. Metabolic syndrome (MetS), characterized by central (abdominal) obesity, insulin resistance, dyslipidemia, and hypertension, constitutes a cluster of risk factors that increase the likelihood of these serious health issues. Metabolic disorders not only place a burden on the cardiovascular system but also exert potentially harmful effects on brain function, which may lead to cognitive impairment and the development of neurodegenerative diseases [1,2,3,4].

One of the key mechanisms linking metabolic disorders to cognitive decline is chronic inflammation. Chronic inflammation, marked by elevated levels of pro-inflammatory cytokines such as interleukin-6 (IL-6) and tumour necrosis factor-alpha (TNF-α), is a hallmark of MetS. These inflammatory markers can cross the blood–brain barrier, potentially leading to neuroinflammation and disruption to brain function, thereby contributing to the pathogenesis of cognitive impairment and neurodegenerative diseases, such as Alzheimer’s disease [5,6,7,8].

Insulin resistance, one of the key components of MetS, is strongly associated with a decline in cognitive function. The mechanisms involve disruption to insulin signalling in the brain, which affects glucose metabolism and synaptic functions [9]. On the other hand, dyslipidemia, characterized by abnormal blood lipid levels, may lead to the formation of atherosclerotic plaques in cerebral vessels, reducing blood flow to the brain and increasing the risk of strokes and dementia [10].

Middle-aged women often experience significant hormonal changes associated with the menopause, which can influence the development of MetS and cognitive function. The decline in estrogen level post-menopause is linked to an increased risk of insulin resistance, dyslipidemia, and central obesity, further increasing the risk of cognitive impairments [11,12]. Estrogen exert neuroprotective effects, and its deficiency may lead to neurodegeneration and cognitive decline [4].

Lifestyle factors, including diet, physical activity, and health habits, play a crucial role in managing both metabolic and cognitive health. A diet high in saturated fats and sugar can contribute to the progress of MetS, whereas a diet rich in fruits, vegetables, fibre, and omega-3 fatty acids may counteract these effects [13,14]. Regular physical activity not only improves metabolic health but also supports cognitive function through neuroprotective mechanisms [15,16]. Physical activity is one of the most effective strategies for moderating the negative consequences of metabolic disorders. Regular exercise can enhance insulin sensitivity, reduce blood pressure, and improve lipid profiles, positively influencing both metabolic and cognitive health [15,17]. Additionally, physical activity promotes the release of neurotrophic factors, such as brain-derived neurotrophic factor (BDNF), which supports neurogenesis and synaptic plasticity, thus offering protection against cognitive decline [18]. Regular exercise can also reduce levels of pro-inflammatory cytokines, further shielding the brain from the detrimental effects of inflammation [19].

Metabolic disorders, such as metabolic syndrome, represent a serious public health problem, especially among middle-aged women. Understanding the mechanisms linking MetS, inflammation, and cognitive function is essential in developing effective intervention strategies. Regular physical activity, a healthy diet, and hormonal regulation can significantly enhance metabolic and cognitive health, reducing the risk of cognitive impairments and improving the quality of life for women of this age. Therefore, the aim of this study was to investigate the association between metabolic disorders, inflammation, and cognitive decline/cognitive functions in a population of middle-aged women.

## 2. Methods

### 2.1. Study Design and Participants

This study used data from two cross-sectional projects involving middle-aged women from the Wielkopolska and Lubuskie Region (Poland). Studies were carried out at the Department of Human Nutrition and Dietetics, Poznań University of Life Sciences, and Department of Dietetics, Faculty of Physical Culture in Gorzow Wielkopolski, Poznań University of Physical Education, Poland. The study protocol was approved by the local ethics committee at Poznań University of Medical Sciences (no. 664/20, 09.09.2020 and no. 989/18, 11.10.2018) in line with the World Medical Association’s Declaration of Helsinki. This study included 179 Polish non-smoking perimenopausal and postmenopausal women (after natural menopause, which means at least twelve months without menstruation or with serum follicle stimulating hormone (FSH) >30 IU/m^2^), aged 43–73 years, without hormone replacement therapy (HTZ). The women were also unable to participate in weight loss projects and did not suffer from chronic systemic diseases such as type 2 diabetes, mental illness, poor communication skills, or other abnormalities that could have influenced the study. Other covariates like age, years of education, physical activity (PA), and cognitive function data were collected through one-on-one interviews conducted by trained research assistants. The study protocol, risks, and benefits were explained to each subject in the study, and written consent to participate in the study was obtained. The study protocol, risks, and benefits were explained to each subject in the study. Written informed consent for participation was obtained from all participants, ensuring compliance with ethical standards. This process was conducted by trained research assistants to guarantee that all participants fully understood the terms of their involvement.

### 2.2. Anthropometry

Anthropometric indices, including height (measured to the nearest 0.1 cm using a stadiometer (RadWag, Poznań, Poland), weight, body composition as the percentage of fat mass and fat-free mass, trunk fat mass (evaluated using the electrical bioimpedance method (BIA) and the TANITA 780 MC analyser) after overnight fasting, waist circumferences (WC), and hip circumference (HC) (measured using non-elastic tape placed horizontally just above the iliac crest with minimal respiration in a standing position (the cut-off was ≥88 cm, suitable for the European women population)) were measured. Body mass index (BMI) was calculated as follows: BMI = weight (kg)/[height (m)]^2^. Women with BMI values < 25 kg/m^2^ were classified as having a normal/correct body weight, those with a BMI score between 25 and 29.9 kg/m^2^ as overweight, and those with a BMI score ≥ 30 kg/m^2^ as obese. WHR was estimated by WC (cm) divided by HC (cm) and WHtR as WC (cm) divided by height (cm). The cut-offs for abdominal obesity using the waist-to-hip ratio (WHR) and waist-to-height ratio (WHtR) were ≥0.8 [World Health Organization. The waist circumference and waist–hip ratio were calculated based on a report from a WHO Expert Consultation, Geneva, 8–11 December 2008, World Health Organization. 2011. [http://www.who.int/nutrition/publications/obesity/WHO_report_waistcircumference_and_waisthip_ratio/en/. accessed on 2 April 2024] and ≥ 0.5 [20,21], respectively.

### 2.3. Clinical and Biochemical Measurements

Systolic and diastolic blood pressure (SBP and DBP, respectively) were measured using 3 consecutive measurements (5 min apart) using a sphygmomanometer with an Omron i-Q142 (HEM-1040-E) arm cuff automatic blood pressure monitor (Omron Healthcare Co. Ltd., Kyoto, Japan) following the standardized criteria of the International Society of Hypertension [22].

Venous blood samples were collected by trained laboratory staff after a 12 h overnight fast. Immediately after blood collection, samples were centrifuged at 1000 rpm for 10 min and stored at −70 °C for analysis. The concentrations of insulin (INS), IL-6, and TNF-α were determined in a serum by the enzyme-linked immunosorbent assay (ELISA) method (Insulin ELISA, IBL International, Hamburg, Germany; TNF-alpha ELISA, DRG Instruments, Marburg, Germany; Quantikine High-Sensitivity IL6 ELISA, R&D Systems, Minneapolis, MN, USA) in line with the manufacturer’s directions. The levels of total cholesterol (TChol), triglycerides (TG), and fasting glucose (GLU) were determined using an automated analyser system (Konelab20i biochemical analyser, ThermoElectron Corporation, Vantaa, Finland). The diagnosis of MetS was established according to the International Diabetes Federation (IDF) criteria. The IDF diagnosis of MetS requires the presence of central obesity (defined based on waist circumference with ethnicity-specific values) or a BMI >30 kg/m^2^ plus any two of the following factors: triglycerides (TG) ≥ 150 mg/dL, blood pressure ≥ 130 (SBP)/85 mm Hg (DBP), fasting glucose (GLU) ≥ 100 mg/dL, or an HDL-C serum concentration < 50 mg/dL for women [International Diabetes Federation. The IDF consensus worldwide definition of the metabolic syndrome. Brussels: IDF, 2006. Available at: https://idf.org/media/uploads/2023/05/attachments-30.pdf/ accessed on 2 April 2024] [23].

The Homeostatic Model Assessment of Insulin Resistance Index was calculated using the following formula: HOMA-IR = [glucose (mmol/L) × insulin (µU/L)]/22.5 OR/and using the HOMA2 Calculator v2.2.3 application [The Oxford Centre for Diabetes. Endocrinology & Metabolism. Diabetes Trial Unit. HOMA2 Calculator: Overview 2018. https://www2.dtu.ox.ac.uk/homacalculator/ accessed on 2 April 2024]. The quantitative insulin sensitivity check index (QUICKI) was used to assess insulin sensitivity using the following formula: 1/[Log Insulin(µU/mL) + Log Glucose (mg/dL)]; the cut-off was 0.34 [24].

### 2.4. Assessment of PA Level

The PA level was determined for all participants by means of the short version of the International Physical Activity Questionnaire (IPAQ-SF). The results were expressed as a low level of PA (<600 metabolic equivalent (MET)/min per week), a moderate level of PA (from 600 to 1499 MET/min per week), or a high level of PA (≥1500 MET/min per week) [25].

### 2.5. Cognitive State Assessment

Cognitive function was examined using the Polish version of the Mini-Mental State Examination (MMSE), the most widely used screening tool for cognitive impairment quantitative assessment. The MMSE is a fully structured, validated scale that consists of thirty points grouped into seven categories: orientation to place, orientation to time, registration, attention, concentration, language, and visual construction [26].

The MMSE test was carried out in accordance with the instructions, taking into account the time limit and the permissible number of three repetitions of each command. The MMSE scale was corrected for age and years of education using the score-adjustment coefficients proposed by Bleecker et al. as follows: MMSE adjusted = MMSE score − (0.471 × (years of education + 12) + (0.131 × (70 − age)). A cut-off score of 26 points in the MMSE test yielded a sensitivity of 43.3% and a specificity of 90.4% for detecting cognitive disorders [27].

### 2.6. Statistical Analysis

All the statistical analysis was performed using Statistica 13.3 (TIBCO Software, Statistica version 13.0; https://docs.tibco.com/products/tibco-statistica-document-management-system-13-3-0, accessed on 2 April 2024) software, with the level of significance set at *p* < 0.05. The Shapiro–Wilk/Kolmogorov–Smirnov test was performed to verify the normality of the distribution of the variables and, as most data were non-normally distributed, nonparametric statistical tests were used. Continuous data are presented as means and standard errors of mean (SEM)/standard deviation (SD) and categorical data as n/%. To compare continuous and categorical variables, women with and without insulin resistant were assessed by HOMA2 IR, the Mann–Whitney U-test, and the Chi^2^ test, respectively. Postmenopausal women with cognitive impairment and women with normal cognitive function were compared in the case of continuous and categorical variables using the Mann–Whitney U-test and the Chi^2^ test, respectively. Groups were compared using these tests regarding anthropometric measurements, metabolic parameters, cognition state, and inflammation. The correlations of serum markers of inflammation with metabolic and cognition status were analyzed using Spearman’s rank correlation coefficient. Unadjusted (Model 1) and adjusted (Model 2—adjusted for age, years of education, BMI and physical activity level) logistic regression models were built to test the relationships between cognition state (MMSEadj scores) and the risk of metabolic disorders, inflammation markers TNF alfa and IL-6 and the odds of the presence of metabolic disorders, and inflammation markers TNF alfa and IL-6 and cognitive impairment (MMSE adj scores). Multivariable logistic regression analysis was used to determine the association between inflammation, metabolic disorders, and cognitive impairment (Model 1—Adjusted for IL6 level and TNF alfa, Model 2—adjustment as in Model 1 plus age and BMI, years of education (continuous), and PA (categorical).

## 3. Results

Table 1 compares groups of women with insulin resistance (*n =* 83) and without insulin resistance (*n* = 96) to assess the impact of inflammation on metabolic disorders and cognitive function. The results show that women with insulin resistance had significantly lower MMSE scores (23.977 ± 3.044 vs. 24.913 ± 2.927, *p* = 0.032) compared to women without insulin resistance. Additionally, the groups differed significantly in terms of age (58.663 ± 6.872 vs. 55.615 ± 6.330, *p* = 0.003), education (14.096 ± 2.681 vs. 15.302 ± 2.318, *p* = 0.006), and metabolic indicators such as body weight (77.683 ± 13.478 kg vs. 70.122 ± 13.810 kg, *p* = 0.000) and BMI (29.673 ± 5.513 kg/m^2^ vs. 26.249 ± 5.096 kg/m^2^, *p* = 0.000).

In the group of women with insulin resistance, a higher percentage of body fat was observed (40.110 ± 6.292% vs. 37.110 ± 7.034%, *p* = 0.005), along with greater lean body mass (45.782 ± 4.738 kg vs. 43.525 ± 5.563 kg, *p* = 0.001) and higher visceral fat content (15.869 ± 6.535 kg vs. 12.633 ± 6.299 kg, *p* = 0.000). The waist circumference (WC) and waist-to-height ratio (WHtR) were significantly higher in the insulin-resistant group (WC, 100.789 ± 12.576 cm vs. 94.554 ± 12.469 cm, *p* = 0.001; WHtR, 0.623 ± 0.085 vs. 0.579 ± 0.079, *p* = 0.000).

Regarding blood pressure parameters, women with insulin resistance had higher systolic blood pressure (SBP, 135.831 ± 19.017 mmHg vs. 127.365 ± 15.209 mmHg, *p* = 0.005), although differences in diastolic blood pressure were not significant (DBP, 85.590 ± 14.039 mmHg vs. 82.542 ± 8.739 mmHg, *p* = 0.293). Glucose levels (GLU, 104.659 ± 21.054 mg/dL vs. 88.899 ± 11.114 mg/dL, *p* = 0.000) and triglycerides (TG, 196.847 ± 113.118 mg/dL vs. 129.425 ± 84.350 mg/dL, *p* = 0.000) were significantly higher in the insulin-resistant group, as was insulin concentration (INS: 18.756 ± 9.723 µIU/mL vs. 8.271 ± 1.195 µIU/mL, *p* = 0.000).

In terms of inflammatory markers, interleukin-6 (IL-6) levels were significantly higher in women with insulin resistance (23.536 ± 110.904 pg/mL vs. 6.145 ± 8.417 pg/mL, *p* = 0.000), although TNF-α levels did not differ significantly between groups (4.770 ± 5.974 pg/mL vs. 4.422 ± 2.343 pg/mL, *p* = 0.513).

Furthermore, a higher percentage of women with insulin resistance met the criteria for metabolic syndrome (67% vs. 46%, *p* = 0.004) and had a BMI ≥ 30 kg/m^2^ (20% vs. 9%, *p* = 0.000), indicating a significant relationship between insulin resistance and the risk of metabolic disorders in this population (Table 1).

Table 2 analyses the correlations between tumour necrosis factor-alpha (TNF-α) concentrations and selected metabolic indicators, as well as cognitive status, in middle-aged women. The results reveal significant correlations between TNF-α levels and cognitive function (assessed using the MMSE scale), glycemia, and body composition.

A negative correlation was observed between TNF-α concentration and MMSE score (*r* = −0.400, *p* = 0.000), suggesting that higher TNF-α levels are associated with cognitive decline. A similar negative correlation was noted with blood glucose levels (GLU) (*r* = −0.268, *p* = 0.000), indicating that elevated TNF-α concentrations are linked to impaired carbohydrate metabolism. The results also demonstrated a significant positive correlation between TNF-α and body fat percentage (FAT%) (*r* = 0.278, *p* = 0.000), suggesting that higher TNF-α levels are associated with increased fat content.

Correlations with other indicators, such as triglyceride levels (TG) (*r* = −0.114, *p* = 0.130) and body mass index (BMI) (*r* = 0.062, *p* = 0.414), were not statistically significant. However, a significant positive correlation between TNF-α and waist circumference (WC) (*r* = 0.200, *p* = 0.008) indicates an association with central fat accumulation.

TNF-α levels were also significantly positively correlated with total cholesterol (TChol) (*r* = 0.217, *p* = 0.004), which may suggest the impact of chronic inflammation on lipid disorders in this group. Other indicators, such as insulin levels (INS) (*r* = −0.068, *p* = 0.367), insulin resistance (HOMA-IR) (*r* = −0.098, *p* = 0.192), and fat-free mass (FFM) (*r* = −0.133, *p* = 0.076), did not show significant correlations with TNF-α levels (Table 2).

Table 3 analyses the relationship between inflammatory markers (TNF-α and IL-6) and the risk of metabolic disorders in middle-aged women using logistic regression models (Model 1 and Model 2). The results presented in Table 3 indicate that TNF-α concentration was not significantly associated with the risk of metabolic syndrome (MetS), general obesity, or hypertension in any of the models. However, a significant association between TNF-α and abdominal obesity, expressed as the waist-to-height ratio (WHtR), was observed in Model 2 (OR = 1.429, 95% CI: 1.005–2.031, *p* = 0.047).

For the IL-6 marker, higher concentrations were significantly associated with hypertriglyceridemia (Model 1, OR = 1.095, 95% CI: 1.053–1.139, *p* = 0.000; Model 2, OR = 1.096, 95% CI: 1.044–1.151, *p* = 0.000) and insulin resistance (HOMA-IR: Model 1, OR = 1.066, 95% CI: 1.028–1.105, *p* = 0.001; Model 2, OR = 1.058, 95% CI: 1.011–1.107, *p* = 0.014). Similar results were obtained for the HOMA2-IR index (Model 1, OR = 1.068, 95% CI = 1.030–1.107, *p* = 0.000; Model 2, OR = 1.059, 95% CI = 1.013–1.108, *p* = 0.012).

The results suggest that while TNF-α is primarily associated with abdominal obesity, IL-6 is a strong predictor of lipid disorders and insulin resistance, indicating different mechanisms by which these cytokines influence metabolic disturbances. Despite the lack of association with metabolic syndrome as a whole, IL-6 shows a stronger relationship with specific components of the syndrome, such as dyslipidemia and insulin resistance, which may point to its more targeted impact on the pathophysiology of metabolic disorders in middle-aged women (Table 3).

Table 4 examines the relationship between inflammatory markers (TNF-α and IL-6) and the risk of cognitive impairments in a population of middle-aged women. The results of logistic regression (Model 1 and Model 2) presented in Table 4 indicate that higher TNF-α concentrations are significantly associated with an increased risk of cognitive impairments. In Model 1, the odds ratio (OR) was 1.382 (95% CI = 1.195–1.598, *p* = 0.000), while in Model 2, after adjusting for additional covariates, the OR was 1.362 (95% CI = 1.153–1.610, *p* = 0.000).

For interleukin-6 (IL-6), no significant relationship was observed between its concentration and the risk of cognitive impairments in any model (Model 1, OR = 1.000, 95% CI = 0.996–1.004, *p* = 0.931; Model 2, OR = 1.002, 95% CI = 0.997–1.008, *p* = 0.370).

These findings suggest that TNF-α may play a key role in the pathogenesis of cognitive impairments in middle-aged women, whereas IL-6 does not appear to be a strong predictor of cognitive decline in this population. Elevated TNF-α levels are associated with a significantly higher risk of cognitive impairments, highlighting the potential significance of this biomarker as an inflammatory marker affecting neuropsychological health in the study group (Table 4).

## 4. Discussion

In this study, women with insulin resistance had significantly higher fasting glucose and insulin levels compared to the non-insulin-resistant group (mean, 104.66 mg/dL vs. 88.90 mg/dL and 18.76 µIU/mL vs. 8.27 µIU/mL; *p* < 0.001). These findings are consistent with data published by Zhang et al. (2020), who showed that those with elevated HOMA-IR exhibited higher fasting glucose and insulin concentrations, contributing to an increased risk of developing type 2 diabetes [28]. Similar results were observed by Kern et al. (2001) and Uysal et al. (1998), who found that insulin resistance was closely associated with deteriorated glycemic profiles and elevated levels of IL-6 and TNF-α [29,30].

In our analysis, women with insulin resistance had higher values for BMI (29.67 kg/m^2^ vs. 26.25 kg/m^2^) and waist circumferences (100.79 cm vs. 94.55 cm). These results are consistent with the findings of Bastard et al. (2006), who showed that central obesity is a key risk factor in the development of insulin resistance and components of metabolic syndrome, including hypertension and dyslipidemia [31]. The higher WHtR values observed in the current study (0.623 vs. 0.579) further confirm these observations, highlighting a clear relationship between abdominal obesity and metabolic disturbances.

IL-6 concentrations were significantly higher in the insulin resistance (IR) group (23.54 pg/mL vs. 6.15 pg/mL; *p* < 0.001), similar findings were presented by Hotamisligil et al. (2006), who described IL-6 as a key pro-inflammatory marker influencing the development of insulin resistance and central obesity [30]. Similar relationships were demonstrated by Palermo et al. (2024), indicating that elevated IL-6 levels are associated with poorer metabolic outcomes and higher cardiovascular risk in individuals with metabolic syndrome [32].

For TNF-α, although the mean concentrations were higher in the IR group, the differences did not reach statistical significance (*p* = 0.513). Conversely, a study by Liu et al. (2016) showed that higher TNF-α levels were linked to an increased risk of diabetes and poorer performance in cognitive tests, suggesting a potential impact of TNF-α on brain health in people with metabolic disorders [33].

The mean MMSE scores in the insulin-resistant group were significantly lower (23.98 vs. 24.91; *p* = 0.032), consistent with the findings of Kim et al. (2023) and Maggi et al. (2009), who also observed that individuals with insulin resistance showed poorer performance in cognitive tests [34,35]. Similarly, Kullmann et al. (2016) and Maggi et al. (2009) demonstrated that insulin action in the brain affects both peripheral metabolism and cognitive function [36]. Furthermore, insulin resistance may serve as an early marker of metabolic disturbances, which over time are significant predictors of cognitive decline in middle-aged populations [37].

In this study, 67% of women with insulin resistance met the criteria for metabolic syndrome compared to 46% of healthy women. These findings are similar to those in studies published by Rutter et al. (2005) and Oprescu (2023); they found that individuals with insulin resistance were at greater risk of developing MetS, as evidenced by higher levels of cholesterol, triglycerides, and fasting insulin [38,39].

In this study, TNF-α concentrations showed a strong negative correlation with MMSE scores (*r* = −0.400, *p* < 0.001). Similar results were reported by Karoly et al. (2021), who examined the relationship between inflammatory markers and cognitive function in older adults [40]. Another study identified TNF-α as a major factor associated with decreased MMSE scores, suggesting that chronic inflammation contributes to neurodegeneration and impaired intellectual performance, particularly in individuals with metabolic syndrome [41]. These findings support the hypothesis that TNF-α disrupts the action of neurotrophins and synaptic processes in the brain, accelerating cognitive decline.

The results in Table 2 indicate a significant negative correlation between TNF-α and glucose levels (r = −0.268, *p* <0.001). This relationship is supported by numerous studies showing that TNF-α blocks the insulin signalling pathway [42,43,44,45,46,47]. Our findings confirm these observations, suggesting that higher TNF-α concentrations are associated with reduced glucose metabolism efficiency.

The results also revealed a significant positive correlation between TNF-α and total cholesterol levels (TChol) (r = 0.217, *p* = 0.004). Similar relationships were observed in studies by Popa et al. (2007), where TNF-α was shown to promote lipolysis in adipose tissue, leading to elevated free fatty acid levels and dyslipidemia [48]. Elevated cholesterol levels may result from chronic inflammation, which increases the production of low-density lipoproteins (LDLs) and contributes to the development of atherosclerosis [49]. These findings suggest that TNF-α may influence lipid abnormalities through its pro-inflammatory action, potentially explaining the increased cardiometabolic risk in individuals with elevated TNF-α levels.

The correlation between TNF-α and fat percentage (FAT%) (*r* = 0.278, *p* < 0.001), as well as waist circumference (*r* = 0.200, *p* = 0.008), highlights its role in promoting abdominal obesity and central fat accumulation. These findings support the literature, where TNF-α is described as a factor inducing localized inflammation in adipose tissue, leading to the increased expression of pro-inflammatory cytokines that collectively enhance adipogenesis [50,51]. Our results suggest that higher TNF-α concentrations may serve as an indicator of excess body fat, particularly visceral obesity, as supported by the study conducted by Kern et al. and Patsalos et al. (2018) [52,53].

Multivariate logistic regression analysis revealed a significant association between TNF-α concentration and the risk of abdominal obesity measured by WHtR (Model 2, OR = 1.429, 95% CI = 1.005–2.031, *p* = 0.047). This result is consistent with findings by Virdis et al. [54], who demonstrated that TNF-α is a key factor promoting fat deposition in the visceral region by increasing the expression of pro-inflammatory markers in adipocytes. Similar findings were reported by Carvalho et al. (2022) [55], showing that higher TNF-α levels correlate with higher WHtR and an increased risk of abdominal obesity.

The role of TNF-α as a promoter of central obesity results from its effects on lipolysis and the inhibition of insulin signalling in adipose tissue, leading to localized inflammation and enhanced fat accumulation. July et al. (2018) described this mechanism as a critical element in the pathogenesis of metabolic syndrome in individuals with obesity. This is consistent with the findings in the current study, where TNF-α was a significant predictor of abdominal obesity but not general obesity [56].

Regarding the IL-6 marker, regression analysis demonstrated a strong positive correlation with the occurrence of hypertriglyceridemia and insulin resistance in both models. The odds ratios for hypertriglyceridemia were OR = 1.095, 95% CI = 1.053–1.139, *p* < 0.001 (Model 1) and OR = 1.096, 95% CI = 1.044–1.151, *p* < 0.001 (Model 2). Similar results were observed for insulin resistance assessed using HOMA2-IR, where IL-6 was significantly associated with the risk of insulin resistance (OR = 1.068, 95% CI = 1.030–1.107, *p* < 0.001).

IL-6 is a strong predictor of lipid disorders and insulin resistance, suggesting diverse mechanisms through which this cytokine influences metabolic disturbances. Despite the lack of association with metabolic syndrome as a whole, IL-6 shows stronger relationships with specific components of the syndrome, such as dyslipidemia and insulin resistance. This indicates its more targeted role in the pathophysiology of metabolic disorders [57] [58,59,60].

Although IL-6 showed strong associations with selected components of metabolic syndrome, it was not significantly correlated with the overall risk of MetS in this study. Similar results were reported by Rehman et al. (2017) and Lehrskov et al. (2019), who demonstrated that IL-6 affected specific components of metabolic syndrome, such as dyslipidemia and insulin resistance, but was not a primary determinant of the development of MetS as a whole [61,62]. This may suggest that IL-6 is a more specific marker for lipid abnormalities and insulin sensitivity rather than a general inflammatory indicator in MetS.

The results of this study suggest that TNF-α may be a strong predictor of cognitive impairments in middle-aged women. Similar relationships were described by Bruunsgaard et al. (1999), who found that elevated TNF-α levels were associated with accelerated cognitive decline in older adults [63]. In their study, TNF-α was strongly correlated with lower scores on cognitive tests and an increased risk of developing dementia, highlighting the impact of inflammation on neuropsychological health [64]. This mechanism may be attributed to the role of TNF-α in microglial activation and the enhancement of neuroinflammatory responses, leading to neuronal damage and impaired synaptic function.

Other studies, such as those conducted by Ahmad et al. (2022), have also shown that chronic inflammation mediated by TNF-α is associated with a higher risk of developing Alzheimer’s disease and other forms of dementia [65]. These findings are similar to our observations, suggesting that TNF-α may serve as an important biomarker for early neurodegenerative changes in individuals with metabolic syndrome and other metabolic disorders.

The results presented in Table 4 support the hypothesis that TNF-α is a significant risk factor for cognitive impairments. Research indicates that TNF-α is one of the main neuroinflammatory mediators, responsible for microglial activation, which leads to the release of additional pro-inflammatory cytokines, such as IL-1β, exacerbating neurodegenerative processes in Alzheimer’s disease [63,65,66,67]. This is consistent with our findings, which show that elevated TNF-α levels are associated with a higher risk of cognitive impairments, potentially reflecting inflammatory processes in the central nervous system.

Our study has certain limitations. The range of measured metabolic parameters and inflammatory markers is limited, which may affect the comprehensiveness of the analysis of inflammatory processes. Specifically, we focused on TNF-α and IL-6, and a wider range could provide a more comprehensive understanding of the inflammatory mechanisms associated with metabolic syndrome. Future research should incorporate longitudinal studies with additional biomarkers (e.g., HbA1C, IL-1, IL-10, CRP, and 2-Hydroxybutyrate). Furthermore, the lack of a longitudinal follow-up prevents the assessment of dynamic changes in inflammatory markers and their impact on cognitive function over time. Despite these limitations, our study provides valuable insights into the associations between inflammatory processes and metabolic disturbances and their potential influence on cognitive function, serving as a foundation for future research in this field.

## 5. Conclusions

The results of this study demonstrate that insulin resistance is closely associated with deteriorated metabolic parameters and cognitive function in middle-aged women. Elevated levels of IL-6 and TNF-α indicate that chronic inflammation may be the key mechanism underlying the development of these disturbances. Comparisons with the literature confirm that insulin resistance is a strong predictor of both metabolic disorders and poorer cognitive test performance.

Our findings confirm that TNF-α is a crucial inflammatory marker influencing metabolic disturbances and cognitive decline in middle-aged women. Its strong correlations with glycemic parameters, body composition, and cholesterol levels suggest a potential role for TNF-α as a therapeutic target in interventions aimed at improving metabolic and cognitive health.

The results of logistic regression analysis indicate that TNF-α is more strongly associated with abdominal obesity, while IL-6 shows stronger connections with insulin resistance and dyslipidemia. Both markers may be key indicators of metabolic disorders, but their specific roles suggest that IL-6 may have greater clinical significance in managing dyslipidemia and insulin resistance in middle-aged women.

Our results suggest that TNF-α, but not IL-6, is strongly linked to the risk of cognitive impairments in middle-aged women, indicating that TNF-α may be a critical marker of early neurodegenerative changes in this group. These findings support the hypothesis that inflammation mediated by TNF-α may accelerate neurodegenerative processes, leading to premature cognitive decline. Further research is needed to determine the molecular mechanisms underlying these relationships and to explore potential therapeutic interventions aimed at reducing inflammation to protect cognitive health.

## Figures and Tables

**Table 1:** Characteristics and distributions by insulin resistance prevalence, presented by HOMA2 IR.

Variables	No Insulin Resistance (*n* = 96)			Insulin Resistance (*n* = 83)			*p*
	Mean	SD	SEM	Mean	SD	SEM	
**MMSE adj**	24.913	2.927	0.321	23.977	3.044	0.311	**0.032**
**Education [y]**	15.302	2.318	0.237	14.096	2.681	0.294	**0.006**
**Age [y]**	55.615	6.330	0.646	58.663	6.872	0.754	**0.003**
**Weight [kg]**	70.122	13.810	1.409	77.683	13.478	1.479	**0.000**
**BMI [kg/m^2^]**	26.249	5.096	0.520	29.673	5.513	0.605	**0.000**
**FAT [%]**	37.110	7.034	0.718	40.110	6.292	0.691	**0.005**
**FFM [kg]**	43.525	5.563	0.568	45.782	4.738	0.520	**0.001**
**Trunk fat [kg]**	12.633	6.299	0.643	15.869	6.535	0.717	**0.000**
**WC [cm]**	94.554	12.469	1.273	100.789	12.576	1.380	**0.001**
WHR	0.910	0.074	0.008	0.925	0.073	0.008	0.232
**WHtR**	0.579	0.079	0.008	0.623	0.085	0.009	**0.000**
**SBP [mmHg]**	127.365	15.209	1.552	135.831	19.017	2.087	**0.005**
DBP [mmHg]	82.542	8.739	0.892	85.590	14.039	1.541	0.293
**GLU [mg/dL]**	88.899	11.114	1.134	104.659	21.054	2.311	**0.000**
**TG [mg/dL]**	129.425	84.350	8.609	196.847	113.118	12.416	**0.000**
TChol [mg/dL]	215.664	43.862	4.477	207.836	46.141	5.065	0.279
**INS [ulU/mL]**	8.271	1.195	0.122	18.756	9.723	1.067	**0.000**
**% B**	106.566	25.389	2.591	138.860	84.351	9.259	**0.000**
**% S**	95.948	15.269	1.558	47.575	15.556	1.707	**0.000**
TNF-α [pg/mL]	4.422	2.343	0.240	4.770	5.974	0.656	0.513
**IL-6 [pg/mL]**	6.145	8.417	0.864	23.536	110.904	12.247	**0.000**
**Presence of MetS [n (%)]**	44 (46)			46 (67)			**0.004**
**BMI < 25 kg/m^2^ [n (%)]**	47 (26)			19 (11)			**0.000**
**25 kg/m^2^ ≤ BMI > 30 kg/m^2^ [n (%)]**	33 (18)			28 (16)			
**BMI ≥ 30 kg/m^2^ [n (%)]**	16 (9)			36 (20)			
PA < 600 MET/min/wk [n (%)]	12 (7)			16 (9)			0.429
PA 600–1500 MET/min/wk [n (%)]	44 (25)			33 (18)			
PA > 1500 MET/min/wk [n (%)]	40 (22)			34 (19)			

Continuous data are presented as means, standard deviation (SD), and standard errors of mean (SEM) and categorical data as n (%). Abbreviations: BMI, body mass index; DBP, diastolic blood pressure; FAT%, percentage of fat mass; FFM, fat-free mass; GLU, fasting glucose; HOMA IR, homeostatic model assessment for insulin resistance; INS, insulin; MET, metabolic equivalent; MMSEadj, Mini-Mental State Examination, adjusted for age and years of education; MetS, metabolic syndrome; PA, physical activity; SBP, systolic blood pressure; TChol, total cholesterol; TG, triglycerides; WHR, waist–hip ratio; WHtR, waist-to-height ratio.

**Table 2:** Correlation of serum inflammation with metabolic and cognition status.

Variables	TNF-α	
	R	*p*
**MMSE adj**	**−0.400**	**0.000**
**GLU [mg/dL]**	**−0.268**	**0.000**
TG [mg/dL]	−0.114	0.130
**Tchol mg/dL**	**0.217**	**0.004**
INS [ulU/mL]	−0.068	0.367
Homa IR	−0.098	0.192
Homa2 IR	−0.092	0.222
BMI	0.062	0.414
**FAT%**	**0.278**	**0.000**
FFM [kg]	−0.133	0.076
**WC [cm]**	**0.200**	**0.008**
SBP [mmHg]	−0.013	0.860
DBP [mmHg]	0.134	0.074

R—Spearman’s correlation coefficient. Abbreviations: BMI, body mass index; DBP, diastolic blood pressure; FAT%, percentage of fat mass; FFM, fat-free mass; GLU, fasting glucose; HOMA IR, homeostatic model assessment for insulin resistance; INS, insulin; MMSEadj, Mini-Mental State Examination, adjusted for age and years of education; MetS, metabolic syndrome; SBP, systolic blood pressure; TChol, total cholesterol; TG, triglycerides; TNF-α, tumour necrosis factor-alpha; WC, waist circumferences.

**Table 3:** Associations between the presence of metabolic disorders and inflammation markers (TNF-α and IL-6).

Variables	Model 1 *				Model 2 **			
	OR	95% CI		*p*	OR	95% CI		*p*
**TNF-α**								
MetS	0.987	0.922	1.056	0.699	0.975	0.897	1.060	0.555
General obesity	0.985	0.912	1.065	0.708	0.959	0.730	1.261	0.766
**Abdominal obesity (WHtR)**	1.239	0.998	1.539	0.052	**1.429**	**1.005**	**2.031**	**0.047**
Hypertension (SBP)	0.965	0.893	1.044	0.373	0.969	0.898	1.046	0.419
Hypertenion (DBP)	0.998	0.933	1.068	0.959	0.994	0.925	1.068	0.867
Hyperglicemia	0.946	0.853	1.049	0.290	0.964	0.877	1.060	0.450
Hypertrigycerydemia	0.899	0.803	1.007	0.066	0.894	0.787	1.014	0.082
Insulin resistance (HOMA IR)	1.017	0.949	1.091	0.627	1.022	0.950	1.100	0.554
Insulin resistance (HOMA2 IR)	1.018	0.950	1.092	0.605	1.023	0.951	1.100	0.543
**IL-6**								
MetS	0.995	0.980	1.011	0.564	0.964	0.920	1.010	0.127
General obesity	0.999	0.993	1.004	0.688	1.001	0.990	1.013	0.845
Abdominal obesity (WHtR)	1.031	0.964	1.102	0.374	1.001	0.985	1.017	0.922
Hypertension (SBP)	0.997	0.989	1.005	0.488	0.979	0.941	1.019	0.307
Hypertenion (DBP)	0.991	0.960	1.023	0.581	0.972	0.934	1.013	0.176
Hyperglicemia	1.000	0.995	1.004	0.850	0.998	0.992	1.004	0.591
**Hypertrigycerydemia**	**1.095**	**1.053**	**1.139**	**0.000**	**1.096**	**1.044**	**1.151**	**0.000**
**Insulin resistance (HOMA IR)**	**1.066**	**1.028**	**1.105**	**0.001**	**1.058**	**1.011**	**1.107**	**0.014**
**Insulin resistance (HOMA2 IR)**	**1.068**	**1.030**	**1.107**	**0.000**	**1.059**	**1.013**	**1.108**	**0.012**

* Model 1—Unadjusted model. ** Model 2—Adjusted for age, years of education, BMI, and physical activity level. Abbreviations: BMI, body mass index; CI, confidence interval; DBP, diastolic blood pressure; HOMA IR, homeostatic model assessment for insulin resistance; MetS, metabolic syndrome; OR, odds ratio; SBP, systolic blood pressure; WHtR, waist-to-height ratio.

**Table 4:** Associations between the occurrence of cognitive impairment and inflammation markers (TNF-α and IL-6).

	Model 1 *				Model 2 **			
	OR	95% CI		*p*	OR	95% CI		*p*
Cognitive impatiment n = 118								
**TNF-α**	**1.382**	**1.195**	**1.598**	**0.000**	**1.362**	**1.153**	**1.610**	**0.000**
IL6	1.000	0.996	1.004	0.931	1.002	0.997	1.008	0.370

* Model 1—Unadjusted model. ** Model 2—Adjusted for age and BMI, years of education (continuous), and PA (categorical). Abbreviations: CI, confidence interval; IL-6, interleukin-6; OR, odds ratio; TNF-α, tumour necrosis factor-alpha.

## Data Availability

The data presented in this study are available on request from the corresponding author.

## References

[1] Saklayen M.G. (2018). The Global Epidemic of the Metabolic Syndrome. Curr. Hypertens. Rep..

[2] Samson S.L., Garber A.J. (2014). Metabolic Syndrome. Endocrinol. Metab. Clin. N. Am..

[3] Kassi E., Pervanidou P., Kaltsas G., Chrousos G. (2011). Metabolic Syndrome: Definitions and Controversies. BMC Med..

[4] Yaffe K., Kanaya A., Lindquist K., Simonsick E.M., Harris T., Shorr R.I., Tylavsky F.A., Newman A.B. (2004). The Metabolic Syndrome, Inflammation, and Risk of Cognitive Decline. JAMA.

[5] Mohammadi M., Gozashti M.H., Aghadavood M., Mehdizadeh M.R., Hayatbakhsh M.M. (2017). Clinical Significance of Serum IL-6 and TNF-α Levels in Patients with Metabolic Syndrome. Rep. Biochem. Mol. Biol..

[6] He Q., Dong M., Pan Q., Wang X., Guo L. (2019). Correlation between Changes in Inflammatory Cytokines and the Combination with Hypertension in Patients with Type 2 Diabetes Mellitus. Minerva Endocrinol..

[7] Yang J., Ran M., Li H., Lin Y., Ma K., Yang Y., Fu X., Yang S. (2022). New Insight into Neurological Degeneration: Inflammatory Cytokines and Blood–Brain Barrier. Front. Mol. Neurosci..

[8] Ogunmokun G., Dewanjee S., Chakraborty P., Valupadas C., Chaudhary A., Kolli V., Anand U., Vallamkondu J., Goel P., Paluru H.P.R. (2021). The Potential Role of Cytokines and Growth Factors in the Pathogenesis of Alzheimer’s Disease. Cells.

[9] Rasgon N.L., Kenna H.A., Wroolie T.E., Kelley R., Silverman D., Brooks J., Williams K.E., Powers B.N., Hallmayer J., Reiss A. (2011). Insulin Resistance and Hippocampal Volume in Women at Risk for Alzheimer’s Disease. Neurobiol. Aging.

[10] Biessels G.J., Staekenborg S., Brunner E., Brayne C., Scheltens P. (2006). Risk of Dementia in Diabetes Mellitus: A Systematic Review. Lancet Neurol..

[11] Brinton R.D. (2008). The Healthy Cell Bias of Estrogen Action: Mitochondrial Bioenergetics and Neurological Implications. Trends Neurosci..

[12] Skoczek-Rubińska A., Chmurzynska A., Muzsik-Kazimierska A., Bajerska J. (2021). The Association Between Fat Taste Sensitivity, Eating Habits, and Metabolic Health in Menopausal Women. Nutrients.

[13] Laitinen M.H., Ngandu T., Rovio S., Helkala E.-L., Uusitalo U., Viitanen M., Nissinen A., Tuomilehto J., Soininen H., Kivipelto M. (2006). Fat Intake at Midlife and Risk of Dementia and Alzheimer’s Disease: A Population-Based Study. Dement. Geriatr. Cogn. Disord..

[14] Cassidy A., O’Reilly É.J., Kay C., Sampson L., Franz M., Forman J.P., Curhan G., Rimm E.B. (2011). Habitual Intake of Flavonoid Subclasses and Incident Hypertension in Adults. Am. J. Clin. Nutr..

[15] Ngandu T., Lehtisalo J., Solomon A., Levälahti E., Ahtiluoto S., Antikainen R., Bäckman L., Hänninen T., Jula A., Laatikainen T. (2015). A 2 Year Multidomain Intervention of Diet, Exercise, Cognitive Training, and Vascular Risk Monitoring versus Control to Prevent Cognitive Decline in at-Risk Elderly People (FINGER): A Randomised Controlled Trial. Lancet.

[16] Middleton L.E., Barnes D.E., Lui L.-Y., Yaffe K. (2010). Physical Activity over the Life Course and Its Association with Cognitive Performance and Impairment in Old Age. J. Am. Geriatr. Soc..

[17] Pedersen B.K., Saltin B. (2015). Exercise as Medicine—Evidence for Prescribing Exercise as Therapy in 26 Different Chronic Diseases. Scand. J. Med. Sci. Sports.

[18] Erickson K.I., Voss M.W., Prakash R.S., Basak C., Szabo A., Chaddock L., Kim J.S., Heo S., Alves H., White S.M. (2011). Exercise Training Increases Size of Hippocampus and Improves Memory. Proc. Natl. Acad. Sci. USA.

[19] Barnes D.E., Yaffe K. (2011). The Projected Effect of Risk Factor Reduction on Alzheimer’s Disease Prevalence. Lancet Neurol..

[20] Ma Y.-L., Jin C.-H., Zhao C.-C., Ke J.-F., Wang J.-W., Wang Y.-J., Lu J.-X., Huang G.-Z., Li L.-X. (2022). Waist-to-Height Ratio Is a Simple and Practical Alternative to Waist Circumference to Diagnose Metabolic Syndrome in Type 2 Diabetes. Front. Nutr..

[21] Waist Circumference and Waist-Hip Ratio: Report of a WHO Expert Consultation. https://www.who.int/publications/i/item/9789241501491.

[22] Unger T., Borghi C., Charchar F., Khan N.A., Poulter N.R., Prabhakaran D., Ramirez A., Schlaich M., Stergiou G.S., Tomaszewski M. (2020). 2020 International Society of Hypertension Global Hypertension Practice Guidelines. Hypertension.

[23] HOMA Calculator. https://www.rdm.ox.ac.uk/about/our-clinical-facilities-and-units/DTU/software/homa.

[24] Assessment of Preferred Methods to Measure Insulin Resistance in Asian Patients with Hypertension—PubMed. https://pubmed.ncbi.nlm.nih.gov/33415834/.

[25] Lee P.H., Macfarlane D.J., Lam T.H., Stewart S.M. (2011). Validity of the International Physical Activity Questionnaire Short Form (IPAQ-SF): A Systematic Review. Int. J. Behav. Nutr. Phys. Act..

[26] Folstein M.F., Folstein S.E., McHugh P.R. (1975). “Mini-Mental State”. A Practical Method for Grading the Cognitive State of Patients for the Clinician. J. Psychiatr. Res..

[27] Mackin R.S., Ayalon L., Feliciano L., Areán P.A. (2010). The Sensitivity and Specificity of Cognitive Screening Instruments to Detect Cognitive Impairment in Older Adults With Severe Psychiatric Illness. J. Geriatr. Psychiatry Neurol..

[28] Zhang W., Tang Y., Huang J., Yang Y., Yang Q., Hu H. (2020). Efficacy of Inulin Supplementation in Improving Insulin Control, HbA1c and HOMA-IR in Patients with Type 2 Diabetes: A Systematic Review and Meta-Analysis of Randomized Controlled Trials. J. Clin. Biochem. Nutr..

[29] Kern P.A., Ranganathan S., Li C., Wood L., Ranganathan G. (2001). Adipose Tissue Tumor Necrosis Factor and Interleukin-6 Expression in Human Obesity and Insulin Resistance. Am. J. Physiol. Endocrinol. Metab..

[30] Uysal K.T., Wiesbrock S.M., Hotamisligil G.S. (1998). Functional Analysis of Tumor Necrosis Factor (TNF) Receptors in TNF-Alpha-Mediated Insulin Resistance in Genetic Obesity. Endocrinology.

[31] Bastard J.-P., Maachi M., Lagathu C., Kim M.J., Caron M., Vidal H., Capeau J., Feve B. (2006). Recent Advances in the Relationship between Obesity, Inflammation, and Insulin Resistance. Eur. Cytokine Netw..

[32] Palermo B.J., Wilkinson K.S., Plante T.B., Nicoli C.D., Judd S.E., Kamin Mukaz D., Long D.L., Olson N.C., Cushman M. (2024). Interleukin-6, Diabetes, and Metabolic Syndrome in a Biracial Cohort: The Reasons for Geographic and Racial Differences in Stroke Cohort. Diabetes Care.

[33] Liu C., Feng X., Li Q., Wang Y., Li Q., Hua M. (2016). Adiponectin, TNF-α and Inflammatory Cytokines and Risk of Type 2 Diabetes: A Systematic Review and Meta-Analysis. Cytokine.

[34] Kim A.B., Arvanitakis Z. (2023). Insulin Resistance, Cognition, and Alzheimer Disease. Obes. Silver Spring Md.

[35] Maggi S., Limongi F., Noale M., Romanato G., Tonin P., Rozzini R., Scafato E., Crepaldi G., ILSA Study Group (2009). Diabetes as a Risk Factor for Cognitive Decline in Older Patients. Dement. Geriatr. Cogn. Disord..

[36] Kullmann S., Heni M., Hallschmid M., Fritsche A., Preissl H., Häring H.-U. (2016). Brain Insulin Resistance at the Crossroads of Metabolic and Cognitive Disorders in Humans. Physiol. Rev..

[37] Abbasi F., Robakis T.K., Myoraku A., Watson K.T., Wroolie T., Rasgon N.L. (2023). Insulin Resistance and Accelerated Cognitive Aging. Psychoneuroendocrinology.

[38] Rutter M.K., Meigs J.B., Sullivan L.M., D’Agostino R.B., Wilson P.W. (2005). Insulin Resistance, the Metabolic Syndrome, and Incident Cardiovascular Events in the Framingham Offspring Study. Diabetes.

[39] Oprescu A.C., Grosu C., Bild W. (2023). Correlations between Cognitive Evaluation and Metabolic Syndrome. Metabolites.

[40] Karoly H.C., Skrzynski C.J., Moe E., Bryan A.D., Hutchison K.E. (2021). Investigating Associations Between Inflammatory Biomarkers, Gray Matter, Neurofilament Light and Cognitive Performance in Healthy Older Adults. Front. Aging Neurosci..

[41] Magalhães C.A., Ferreira C.N., Loures C.M.G., Fraga V.G., Chaves A.C., Oliveira A.C.R., de Souza L.C., Resende E.d.P.F., Carmona K.C., Guimarães H.C. (2018). Leptin, hsCRP, TNF-α and IL-6 Levels from Normal Aging to Dementia: Relationship with Cognitive and Functional Status. J. Clin. Neurosci..

[42] Hotamisligil G.S. (1999). Mechanisms of TNF-α-Induced Insulin Resistance. Exp. Clin. Endocrinol. Diabetes.

[43] He M.M., Smith A.S., Oslob J.D., Flanagan W.M., Braisted A.C., Whitty A., Cancilla M.T., Wang J., Lugovskoy A.A., Yoburn J.C. (2005). Small-Molecule Inhibition of TNF-Alpha. Science.

[44] McMillan D., Martinez-Fleites C., Porter J., Fox D., Davis R., Mori P., Ceska T., Carrington B., Lawson A., Bourne T. (2021). Structural Insights into the Disruption of TNF-TNFR1 Signalling by Small Molecules Stabilising a Distorted TNF. Nat. Commun..

[45] Leung C.-H., Zhong H.-J., Yang H., Cheng Z., Chan D.S.-H., Ma V.P.-Y., Abagyan R., Wong C.-Y., Ma D.-L. (2012). A Metal-Based Inhibitor of Tumor Necrosis Factor-α. Angew. Chem. Int. Ed..

[46] Alonso-Castro A.J., Zapata-Bustos R., Gómez-Espinoza G., Salazar-Olivo L.A. (2012). Isoorientin Reverts TNF-α-Induced Insulin Resistance in Adipocytes Activating the Insulin Signaling Pathway. Endocrinology.

[47] Akash M.S.H., Rehman K., Liaqat A. (2018). Tumor Necrosis Factor-Alpha: Role in Development of Insulin Resistance and Pathogenesis of Type 2 Diabetes Mellitus. J. Cell. Biochem..

[48] Popa C., Netea M.G., Van Riel P.L.M., Van Der Meer J.W., Stalenhoef A.F. (2007). The Role of TNF-α in Chronic Inflammatory Conditions, Intermediary Metabolism, and Cardiovascular Risk. J. Lipid Res..

[49] Tsoupras A., Lordan R., Zabetakis I. (2018). Inflammation, Not Cholesterol, Is a Cause of Chronic Disease. Nutrients.

[50] Sethi J.K., Hotamisligil G.S. (2021). Metabolic Messengers: Tumour Necrosis Factor. Nat. Metab..

[51] Hildebrandt X., Ibrahim M., Peltzer N. (2023). Cell Death and Inflammation during Obesity: “Know My Methods, WAT(Son)”. Cell Death Differ..

[52] Kern L., Mittenbühler M.J., Vesting A.J., Ostermann A.L., Wunderlich C.M., Wunderlich F.T. (2018). Obesity-Induced TNFα and IL-6 Signaling: The Missing Link between Obesity and Inflammation—Driven Liver and Colorectal Cancers. Cancers.

[53] Patsalos O., Dalton B., Leppanen J., Ibrahim M., Himmerich H. (2020). Impact of TNF-α Inhibitors on Body Weight and BMI: A Systematic Review and Meta-Analysis. Front. Pharmacol..

[54] Virdis A., Colucci R., Bernardini N., Blandizzi C., Taddei S., Masi S. (2019). Microvascular Endothelial Dysfunction in Human Obesity: Role of TNF-α. J. Clin. Endocrinol. Metab..

[55] Carvalho W.R.C., França A.K.T.d.C., Alcione A.M.D.S., Padilha L.L., Bogea E.G. (2022). Waist-to-Height Ratio Cut-off Points to Predict Obesity in Adolescents and Associa-Tion with Inflammatory Markers. Nutr. Hosp..

[56] July M., Faiz S., Yaqub A., Santhanam P., Douglas J., Stanek R., Gress T., Olajide O., Driscoll H., Santanam N. (2018). Role of Adipokines and Inflammatory Markers in Postmenopausal Hypertension. Minerva Endocrinol..

[57] Yan J., Yang S., Han L., Ba X., Shen P., Lin W., Li T., Zhang R., Huang Y., Huang Y. (2023). Dyslipidemia in Rheumatoid Arthritis: The Possible Mechanisms. Front. Immunol..

[58] Du Y., Zhang Q., Zhang X., Song Y., Zheng J., An Y., Lu Y. (2023). Correlation between Inflammatory Biomarkers, Cognitive Function and Glycemic and Lipid Profiles in Patients with Type 2 Diabetes Mellitus: A Systematic Review and Meta-Analysis. Clin. Biochem..

[59] Toader M.P., Taranu T., Constantin M.M., Olinici D., Mocanu M., Costan V.V., Toader S. (2021). High Serum Level of Interleukin-6 Is Linked with Dyslipidemia in Oral Lichen Planus. Exp. Ther. Med..

[60] Nisar M., Iqbal M.-U.-N. (2021). Influence of Hs-CRP, IL-6 and TNF-α and It’s Role in Dyslipidemia and Type 2 Diabetes in Population of Karachi, Pakistan. Pak. J. Pharm. Sci..

[61] Rehman K., Akash M.S.H., Liaqat A., Kamal S., Qadir M.I., Rasul A. (2017). Role of Interleukin-6 in Development of Insulin Resistance and Type 2 Diabetes Mellitus. Crit. Rev. Eukaryot. Gene Expr..

[62] Lehrskov L.L., Christensen R.H. (2019). The Role of Interleukin-6 in Glucose Homeostasis and Lipid Metabolism. Semin. Immunopathol..

[63] Plantone D., Pardini M., Righi D., Manco C., Colombo B.M., De Stefano N. (2023). The Role of TNF-α in Alzheimer’s Disease: A Narrative Review. Cells.

[64] Bruunsgaard H., Andersen-Ranberg K., Jeune B., Pedersen A.N., Skinhøj P., Pedersen B.K. (1999). A High Plasma Concentration of TNF-α Is Associated With Dementia in Centenarians. J. Gerontol. Ser. A.

[65] Ahmad M.A., Kareem O., Khushtar M., Akbar M., Haque M.R., Iqubal A., Haider M.F., Pottoo F.H., Abdulla F.S., Al-Haidar M.B. (2022). Neuroinflammation: A Potential Risk for Dementia. Int. J. Mol. Sci..

[66] Jayaraman A., Htike T.T., James R., Picon C., Reynolds R. (2021). TNF-Mediated Neuroinflammation Is Linked to Neuronal Necroptosis in Alzheimer’s Disease Hippocampus. Acta Neuropathol. Commun..

[67] Rauf A., Badoni H., Abu-Izneid T., Olatunde A., Rahman M.M., Painuli S., Semwal P., Wilairatana P., Mubarak M.S. (2022). Neuroinflammatory Markers: Key Indicators in the Pathology of Neurodegenerative Diseases. Molecules.

